# The Rough Road: A Single Case Study of Dreamtelling in a Group during the COVID-19 Pandemic and Military Conflict

**DOI:** 10.3390/ijerph19127174

**Published:** 2022-06-11

**Authors:** Shulamit Geller, Robi Friedman, Sigal Levy, Yehoshua Akerman, Gal Van den Brink, Guy Romach, Tuli Shazar, Gil Goldzweig

**Affiliations:** 1 School of Behavioral Sciences, The Academic College of Tel Aviv-Yaffo, Tel-Aviv 68182, Israel; josh242@gmail.com (Y.A.); galvandenbrink1@gmail.com (G.V.d.B.); romach.g@gmail.com (G.R.); shazartuli@gmail.com (T.S.); giligold@mta.ac.il (G.G.); 2 International Group Analytic Society, Israeli Institute of Group Analysis, Haifa 33095, Israel; robifriedman@gmail.com; 3 Statistical Education Unit, The Academic College of Tel Aviv-Yaffo, Tel-Aviv 68182, Israel; levy@mta.ac.il

**Keywords:** dreaming, COVID-19, lockdown, group work, dreamtelling

## Abstract

Sharing dreams is a common practice, and several motives, such as emotional processing, emotional relief, and request for containment, have been identified. An exploratory single case study research design was used to explore the experiences of the COVID-19 pandemic and local military conflict among a group of Israeli students. The group discussed a dream previously shared in social network sites during the first COVID-19 lockdown. A qualitative content analysis of the meeting transcript yielded three meaningful and coherent themes: feeling blocked and helpless in front of a barrier; a sense of intrusion, defense, and psychological coping; belonging to the group as a means of coping with an individual and a collective threat. Each of these themes reflected personal, interpersonal, and social aspects of the participants’ experiences. The results deepen the understanding of people’s dominant experiences and main psychological coping mechanisms during a collective stressful event. Further, they support the positive effect of the dreamtelling approach on individuals’ coping experiences and on enhancing hope by sharing and discussing dreams with others.

## 1. Introduction

### 1.1. Dreaming during Traumatic and Stressful Events

COVID-19 has been found associated with a notable increase in both individual and community psychological distress worldwide on both national and international levels, [1]. The threats of dying of an unfamiliar disease, infecting loved ones, and suffering financial loss were extenuated by the measures taken against mass infection such as quarantine and social isolation. Studies reported overt negative psychological effects, including post-traumatic stress symptoms, confusion, and anger [2]. Recent studies have shown that this period, such as other periods of collective distress [3], had a strong impact on dreaming [4]. The stressful experiences led to the rise of numerous websites that collected dream reports and contents during the first lockdown period (March–May 2020) and received broad worldwide press coverage.

### 1.2. The Function of Dreaming

Psychoanalytic theories and practices beginning with Freud [5] share the assumption that dreams play a role as mental mechanisms which deal with excessively emotional experiences and effects. This idea was further developed by other theoreticians, such as Bion [6], who conceptualized the dream as a “container,” thus allowing one to process material that, prior to dreaming, was unthinkable. 

Contemporary dream theory and research have argued that, in times of continuous stress, dreaming may help people to cope with negative emotions and distress [7,8,9]. It may, at the same time, open additional adaptive mental functioning for emotional processing in which neural connections are formed more easily than in a waking state [10]. These connections are, thus, not made randomly nor do they mirror actual traumatic experiences but, rather, are guided by the dreamer’s emotions. Hartmann [11] referred to this connection making as “weaving in” material, connecting stressful experiences and emotions with existing memory, thus making possible the “calming of the storm”. Recent research findings of investigations of COVID-19 pandemic lockdown dreams in Italy revealed that focusing on the function of dreams during such traumatic events facilitated the affective integration and elaboration [12] of difficult emotional contents. 

### 1.3. Dreams Shared in Group: The Group Analytic Approach 

Dream sharing and interpretation has its roots in ancient human history [13]. Across human evolution, the fictional and story-like aspects of dreams have been shared around campfires to help understand causality [14] as well as to enhance learning, bonding, and corrective mechanisms [15]. It has been argued that social network sites (SNSs) that enabled individuals to share their frequent, vivid, bizarre, and emotionally intense dreams during the COVID-19 pandemic [4] replaced the tribal campfire, while allowing individuals and communities around the world to exchange informational and emotional support during quarantine and social isolation [15,16]. 

From a group analytic perspective, SNSs represent a web of relationships in which each item or individual is linked to another, serving as a nodal point and leading to other links or nodal points, all interconnected together in a deeper matrix [17]. The *matrix*—a key concept in group analysis—is the common ground of virtually all communications (conscious and unconscious, past and present), the sociocultural organization, and the context [18]. This context includes the organization of the wider society within which the group operates, which is termed the “foundation matrix” and includes elements such as language, social status, and historical narratives and traditions [19]. The “dynamic matrix” that emerges from the interaction of individuals with the foundation matrix [20] is the organization of the group itself and is based on the interrelations of its patterns of communication. The relations between the dynamic and foundation matrices are reciprocal; all communications lend meanings to the group process [21]. The dreamer and their listeners meet in the dynamic matrix (e.g., the SNS dream-sharing group) in which the dream is both an individual creation and the property of the group, namely, a co-creation that is analyzed by all members through their reflections and occurrences in the group [22]. In line with this conceptualization, the dream responds to a collective concern in the matrix, while the dreamer may be considered as having a delegated role [23] as the one who initiated its elaboration. When the dream is shared in the group, other members of the collective become involved in this process of containment and elaboration. 

In the context of contemporary group theories, dreaming and dreamtelling are perceived as communications with a “request for containment” [24], necessary for stimulating new ways of thinking and understanding disavowed personal, social, and cultural experiences [25,26,27]. Sharing and discussing dreams in a group setting may, thus, expand members’ empathy [28] and ability to deal with unresolved stressful and traumatic collective experiences as a form of social and “moral witnessing” [29]. Specifically, Bermundez [30] used illustrations in his case study to advocate for the importance of such witnessing in containing and achieving a reflective capacity for unformulated and non-symbolized collective traumatic experiences. In accordance with these results, a recent study on pandemic dreams [31] found that while these dreams reflected mental suffering, the process of observing and reporting them was positively evaluated by participants. These findings indicate that telling, sharing, and talking about dreams during social isolation can have a positive effect on individuals’ experiences, helping them to mitigate mental suffering and potentially leading them to engage in other forms of creative social connection. 

When applying the dreamtelling approach to the first COVID-19 lockdown and SNSs, four stages may be assumed [25]. In the first stage, the shock of COVID-19—a once-in-a-lifetime traumatic event and a threat for the individual and the community—concerns groups at both national and international levels. This universal threat is then dreamed by the dreamer in a second stage, after which the dreamer remembers the dream and self-reflects on it in a third stage. In the fourth stage, when the “digestion” is not sufficient, the dream is written and sent to the web where the joint processing of the dream narrative and its resonance is made possible. This digestion, thus, goes beyond the individual to be processed “among partners”, and the dream and, hence, the whole process, are “re-dreamed” by the listeners.

### 1.4. Israeli Social Reality May 2021–June 2021

May 2021 was marked in Israel by a series of riots and violent conflicts between Arabs and Jews in mixed Israeli cities. The riots broke out against the background of Israeli Army airstrikes in Gaza Strip, which resulted in missiles attacks from Gaza to Israel. One focus of the riots was the area surrounding Academic College of Tel Aviv–Yaffo where both Arab and Jewish students study. Although there were no riots in the college, there was a substantial amount of violence in the neighboring streets. The college, which had just reopened after the end of the second wave of COVID-19, had to be closed for another few days. 

As with many traumatic events, once the college reopened after the ceasefire, there was a dissociation between the factual and the emotional memories of the events [32]. Students reported elevated anxiety about returning to the campus and to life in general and found it difficult to connect with the associated shock, anxiety, and terror.

Considering the adaptive function of dreaming [33,34] and the assumption that dreams have the potential to contribute to our understanding of the social and institutional reality of individuals [20,26], the present case study is a novel attempt. It uses a dream posted and shared on SNSs during COVID-19 to describe how a single one-time dreamtelling session among a group of students may reveal unique themes regarding both individual and social experiences during stressful events and enhance psychological coping mechanisms.

## 2. Methodology 

### 2.1. Study Design

The current study followed a qualitative single case study research design which enables an in-depth examination of unique events occurring at a specific point in time and the collection of rich and naturalistic data of collective processes, individual experiences, and psychological coping mechanisms to be performed [35,36]. This approach was used to examine the case of dreamtelling in a group during the stressful events of both the COVID-19 pandemic and military conflict.

### 2.2. Participants and Procedure

Advertisements recruiting participants to the dreamtelling group were placed on the website of Academic College of Tel Aviv–Yaffo and other student social media platforms (e.g., WhatsApp groups) following the COVID-19 lockdown and the military conflict. The final group comprised ten participants who responded to the advertisement: two undergraduate students, five graduate students, and three alumni of the college’s School of Behavioral Sciences. All participants were Jewish Israeli, comprising five men and five women. The average age range was 22–32 years old. 

### 2.3. Data Collection

The dreamtelling single group session took place at the college for 90 min in May 2021 and was conducted by the first and second authors. It opened with a presentation of the “rough road” dream (see below) that was shared in a designated Facebook group called “350 Dreamers” in March 2020 during the first worldwide lockdown. It was selected randomly for the present case study with the inclusion criterion of being relatively abstract and not explicitly related to the pandemic. The session was conducted according to a dreamtelling protocol, aimed to structure a discussion among participants. Participants were informed that participation was voluntary and that the session would be transcribed by a research assistant who was also a participant in the group without giving any identifying information. 

#### Dreamtelling Protocol

- Write down the dream in your own words;
- Choose two meaningful words and write down your associations with them (spend one and half minutes on each word);
- Share your associations with these words;
- Write down what is “of me” in the dream and what is “for me” in the dream;
- Share the personal resonances and memories evoked by the previous stages.

### 2.4. Data Analysis

The study design is based on a qualitative single case study of a particular group session as a one-time unique event, using an in-depth qualitative content analysis approach to process the data of the group as a whole [20]. First, we systematically managed the documentation of the dreamtelling group session. Second, we familiarized ourselves with the transcription through repeated readings. Third, we extracted codes from the data and used them as the basis for creating categories and sub-categories (see Appendix A). Finally, we formulated three main themes [36,37].

### 2.5. The Rough Road Dream

The Rough Road Dream is quoted verbatim as it was shared in the Facebook group “350 Dreamers” in March 2020. In addition, the quotes in the following section were all translated from the original Hebrew by the authors:

*I am driving my somewhat worn-out Matrix up a rough dirt road I have been on before and come to a place where I have to go over a low but steep rise to access the main road. There are large boulders/rocks/earth, which have not been there previously, in the path of the exit location, preventing my exit. I start to drive around this and find large piles of rough earth have been placed at and around the exit, preventing easy access to the main road. I drive the car slowly to the right and while very awkward, I make it over the rough earth.*

## 3. Results

Content analysis of the meeting transcript yielded three meaningful and coherent themes: (1) feeling blocked and helpless in the face of a barrier; (2) a sense of intrusion, defense, and psychological coping; and (3) belonging to the group as a means of coping with an individual and a collective threat. The recent military conflict resonated in each of the three themes. 

We also identified a continuous dialectical movement between the individual and the group. This was reflected in the tension between the general and collective dream contents and more personal associative contents. The following description of the themes relates to both the military conflict and this dialectical movement.

### 3.1. Feeling Blocked and Helpless in the Face of a Barrier

The explicit story of the “rough road” dream shows that the person in the dream succeeded in overcoming the barrier being faced. Nonetheless, the participants referred to negative feelings and fears related to being blocked and feeling helpless in front of that barrier.

*I’m already there and I want to move forward, but it is blocked and I can’t get passed it.*

*(Male graduate student)*

*An experience of helplessness…something that wasn’t there before and suddenly emerges unintentionally.*

*(Male undergraduate student)*

While at the beginning of the session the experience of being blocked and helpless was associated with the dream content and with the general sense of threat and the accompanying restrictions, it was later expressed as a personal feeling shared in the group.

*I was filled with anxiety and I couldn’t free myself from a state of “what will they think of me” and how will I be.*

*(Female graduate student)*

This experience of being observed by others may have reflected the participants’ anxiety as well as the effort to “belong”, which characterizes early stages of group development [38]. It may, therefore, be argued that this inevitable anxiety was exacerbated in the present group whose aim related to participants’ concerns during stressful times.

One participant expressed a wish to move on to a new place.

*I am stuck in a familiar place and I want to change places, I want to move on but I can’t, I am stuck.*

*(Male undergraduate student)*

He, nonetheless, felt helpless and stuck concerning this wish. In the group discussion, stagnation and helplessness were found to be linked with uncertainty and worry about the future several times.

*Recently I went back to my parents’ house and, once again, I found it difficult to take the step of leaving the house and I found it strange that it was difficult again.*

*(Female graduate student)*

*Now I am supposed, perhaps, to open a private practice…to grow or not to grow, can I or can’t I?*

*(College alumna)*

*There’s no future, there’s no place to go, where am I going to? That’s what it reminded me of…like the solution to an infinite equation.*

*(Male graduate student)*

The sense of being blocked was particularly pronounced as participants expressed difficulties relating to the specific personal developmental phases and challenges they faced (e.g., graduation, moving out of their parents’ homes). 

These feelings were also associated with the experience of feeling blocked during the recent military conflict. Importantly, this was addressed vicariously through memories and experiences related to their military service (an integral part of Israeli society).

*I carried a firearm in the army and I was very good at operating it…and suddenly, the feeling in the dream that the muscles aren’t working.*

*(Female graduate student)*

*In the army I had a recurrent dream that I didn’t manage to pull the trigger.*

*(Female undergraduate student)*

These military associations exemplify feelings of stagnation, helplessness, and life- threatening situations. In this context, the participants’ associations express a confrontation with a challenging and long-standing reality of experiencing collective traumas and glories [39]. Ongoing existential threats transform the Israeli soldier’s matrix [40] from a foundation matrix, which is a latent basic disposition in the social unconscious [21], into a manifest and present dynamic matrix, which influences everyone in society.

The flow of the group discussion was, thus, characterized by movement from a general discussion relating to the experience of being stuck, as was apparent in the dream, to a personal experience of being stuck and wishing to be freed from society’s demands and expectations. Furthermore, the sense of helplessness caused by the barrier may be understood as an allusion to the anxiety experienced at the beginning of the session. The possibility to share these feelings with the group provided a containment and an understanding of the individual and social experiences during COVID-19 and the recent military conflict.

### 3.2. Intrusion, Defense, and Psychological Coping 

The aforementioned sense of helplessness was accompanied by strong feelings of intrusion. As shown below, participants were continually searching for mechanisms to help them defend themselves. The somewhat implicit associations with intrusion and, in some cases, with defense against it began with spontaneous recollections and experiences of the recent military conflict and other memories of collective stress in the Israeli context.

*At the time of escalation in Israel and Gaza…there were a lot of demonstrations there…and I can’t get to the sea…. This picture really reminds of that.*

*(Male graduate student)*

It may be argued that being under the threat of rockets, riots, and the inability to maintain one’s routine (e.g., going to the beach) represents feelings of intrusion. The following associations underline the personal connection to the military context.

*I once had a dream that there was a war…and, at the end, they shot me in the dream, it was a really difficult feeling.*

*(Female graduate student)*

There were also very personal associations relating to intrusion and defense.

*When I was living with my parents, I dreamed that someone was trying to break into the house, and I was trying to defend myself and couldn’t.*

*(Female undergraduate student)*

The group session, which began with a general discussion relating to intrusion and defense in times of conflict and threat, thus, became more personal, with participants relating to the issue of feeling secure or protected or being personally intruded on. The main resonating question was who was supposed to protect them and how.

*I felt that things would not be OK…there’s something not very simple going on here, no one is protecting me.*

*(Female undergraduate student)*

*When I was little, I was traveling with my dad…he held me so that I didn’t throw up. In hindsight, I think it would have been better if we had stopped and I had taken a breath of fresh air, but [instead] he held me. I haven’t thought about it for years, it was a really unpleasant experience.*

*(College alumnus)*

In these associations we identified the desire for a parental figure who would hold and protect the participants (e.g., their own parents, group leaders, or national leaders) from external threats such as thieves, enemies, and the pandemic, as they consider their inner resources insufficient. 

Other defense attempts included more implicit coping efforts that were characterized by feelings of happiness (at a wedding) and movement upwards, which may exemplify manic coping mechanisms [41], i.e., mechanisms that involve the denial of psychic reality through omnipotence and triumph over the objects. This defense allows a reduction in feelings such as loss, guilt, and helplessness that emerge in harsh social conditions to be achieved [42].

*I was at a good friend’s wedding and I came home inspired to look for something new.*

*(Female graduate student)*

*It reminded me of an old dream. I dreamed about driving to the top of a mountain…I was in the car on the open road with a far too crazy incline and a very beautiful view opened Infront of me.*

*(Female graduate student)*

*It reminded of a trip… a picture of a stream…with huge rocks on the trail on both sides. I had a warm and positive feeling.*

*(College alumnus)*

Despite the fact that most of the dream associations related to distress, it was evident that, through the presented dream, the participants felt invited to observe, share, play, and engage with the task more willingly [43]. The creativity of associations and the diversity of defense mechanisms employed by the participants demonstrate the dialectical movement between sharing, collectivity, and personal concerns.

### 3.3. Belonging to the Group as a Means of Coping with an Individual and a Collective Threat

Throughout the session, the participants’ wish to relate to each other and to the group as a whole [20] through a more interpersonal and intersubjective discussion was clearly manifested.

*When you walk in the forest, there are threatening boulders but it becomes apparent that they are cute and friendly trolls…partners on the journey.*

*(Male graduate student)*

*It made me think of us here in the group. Maybe we too are slowly becoming subjects.*

*(College alumna)*

The above associations illustrate attempts to transform the obstacle (i.e., the threatening boulders) into an intersubjective experience. As the trolls become “partners on the journey,” the content ceases to be intrapersonal and becomes interpersonal. The concept of the “road” presented in the dream’s title and content thus resonated throughout the group discussion. The transformation of the frightening boulders into cute trolls and partners may be understood as an attempt to associate the boulders with the threat caused by the military conflict while distancing and bypassing the threat as a way of coping with it within the group.

*The comfort of not being alone with it.*

*(Female graduate student)*

*Bypassing it together or looking at it together.*

*(College alumnus)*

The mutual progress of the group as partners on the journey created a sense of comfort among members.

*This collectivity which made me feel more comfortable.*

*(College alumna)*

*The sense of a shared effort…I feel like I was charged with energy and now I feel less tired.*

*(College alumnus)*

Sharing difficult and even traumatic experiences in the group, in contrast with the solitariness of the dreamer of the “rough road,” gave the participants a safe place where they could express their reactions to both the military and pandemic threats. This, in turn, enabled them to feel more vital and less ashamed of asking for help and requesting containment [24].

*What happened in the dream [i.e., overcoming the barrier] also happened in the group. We had to get to this thing together…that the dreamer was alone, asking for help.*

*(Male undergraduate student)*

*I was reminded of Winnie the Pooh when Tigger comes all bouncy to the forest and takes everyone out on a walk and they realize that they are getting lost…walking in circles. Then they want to go home and Piglet says, “But how? We are lost” and Pooh says, “Until now there was noise…but now there is silence I will listen to it and get us home.”*

*(College alumnus)*

This association, which reads as a “story”, demonstrates the group’s narrative in the current stressful situation where the group can provide the means to both find the way (i.e., the way home) and get lost. It also demonstrates the dialectical movement between both quietness and chaos, and the individual and the group.

The process taking place during the session can be seen as a kind of movement that created the group’s dynamic matrix. This matrix enabled the participants to listen to the potential carried by the unspoken communication [44] that touched on the sources of distress located in society and in themselves. In turn, sharing and containing both individual and collective experiences in the face of fear, helplessness, and uncertainty exemplified the support provided by belonging to the group. 

## 4. Discussion

The current case study explored the experiences of the COVID-19 pandemic and a local military conflict among a group of Israeli students. The findings of the study suggest that both the pandemic and military conflict impacted the students. The identified contents reflected helplessness, threat, defense, psychological coping, and personal military experiences. The group process was characterized by dialectical movement between the individual and the group that enabled participants to contain their experiences and enhanced their coping. 

Overall, the study’s first two themes correspond to existing pandemic dream research [45]. Specifically, although not explicitly related to the social context, these themes related to the affective experiences of inefficacy, i.e., feeling blocked and unable to continue, helpless, and under threat. The challenge relating to the mastery of a coping device (e.g., blocked roads, a weapon) may have also represented the participants’ global psychological concerns, i.e., the diminished life opportunities and life plans put on hold during lockdowns with which students were struggling during this period [12,45]. 

Psychoanalytic accounts, e.g., [46], and subsequent trauma research, e.g., [47,48], have described helplessness, avoidance, and intrusion symptoms [49] as characterizing the overwhelming emotional consequences of trauma. It is, thus, not surprising that in the context of the vulnerability and deficiency perceived during both the lockdowns and military conflict these emotions were highly present in participants’ experiences. 

Yet, the ability to share personal ambitions as well as the individual striving for competence and success in the “here and now” of the dynamic matrix, both of which have been found to be typical of psychology students [50], may reflect the students’ coping attempts and the level of hope for their futures that they were able to maintain [51]. Similarly, the presence of associations and dreams related to outdoor/landscape experiences (e.g., the beach, rivers, mountains) may be interpreted as reflecting the participants’ conscious and subconscious need and desire to be released from isolation and screens and return to the pre-apocalyptic “good old days”.

From a complementary angle, the depiction of manic defenses in the present group may be understood as an effort to restore a damaged sense of omnipotence and the guiltlessness of both individuals and their society while denying fear and destructive impulses and wishes [52]. 

### 4.1. Military Context and Metaphor

Memories and experiences relating to personal military service may be understood as a disguise for COVID-19. In medical language and the media, illness is often described using military metaphors [53]. Likewise, COVID-19 was depicted as a “deadly enemy”, with doctors and health professionals being described as “heroes” or “soldiers” “fighting on the front line” in a “battle” against the pandemic and its lethal effects [12]. 

From a group analytic perspective, this use of army associations and the discussion that later developed around the difficulty of pulling the trigger may be understood as expressions of the Israeli soldiers’ matrix [40]. This conceptual framework enables the identification and exploration of the impact of war and other army conditions on a community or society. In the Israeli context, which propagates an ethos of battle glory, its essential meaning is that every civilian, both men and women, is conscripted, and no one is ever truly released from army service. Therefore—and since the social context at the time involved military conflict—the identification with the soldier’s matrix revealed its significance as a template that manifests both trauma, fears, and wishes for glory and thus bestowed value [39]. It may, more specifically, have represented the wish for the group to contain some of these repressed and split personal, interpersonal, and social feelings of fear and aggression and, at the same time, to create a safe place providing reassurance and value.

### 4.2. From SNSs to the Group in Tel Aviv and Back 

From a group analytic perspective in general and the dreamtelling approach [24] in particular, the present case study demonstrates that dreams reflect a collective cultural product and thus can be dreamed for other people with whom we identify or share a concern [25]. Especially during this time of lockdown, loneliness, and intense fear, the use of dreams and the ability to re-dream [22] the dream together allowed the participants to transform implicit material into explicit material [27]. As the unspoken difficulties of the participants’ experiences were exchanged, they found words hitherto not explicitly expressed [44].

The use of an SNS dream served as a kind of playful transitional space [54]—a dream that comes from nowhere and from no one that invites participants to share, play, and travel as “casual tourists” [55]. The renewed dreamtelling process “opened-up” a space that allowed movement between me and not me, us and them, which has been shown to enhance the capacity to symbolize and to transcend the effects of the current collective stressful experience [56]. 

Indeed, this joint digestion of shared difficulties was regarded by all the participants as a creative process that made room for feelings of belonging, quietness, and success as well as feelings of exclusion and failure. This process, thus, helped them transform the initial noise and chaos into new co-constructed meanings [57].

As co-authors and researchers of the present case study, we found ourselves involved in an emotional experience throughout the entire joint project in general and the writing process in particular. Our inability to influence both our and our students’ helplessness and uncertainty stemming from the recent collective stressful events led us to find containment in our belonging to both the dream research group and the dreamtelling group. In our search for wider emotional support and more information, we initiated the process of connecting to the SNS dream matrix by retrieving a dream and then sharing it with a group of students for re-dreaming and dreamtelling. This dual process yielded two main outcomes: (1) the exposure of profound depth structures and hidden themes that could not have been exposed in any other way in such a short time and (2) the digestion and relief of mental pain through the creative social connections forged within the group. Moreover, this unique social and psychological safe space for sharing and imagining enabled both researchers and students to discover new multipath routes to themselves and back to the wider society that enhanced the development and maintenance of hopeful attitudes for others as well as for oneself [56]. Such attitudes initiated the creative process of writing this paper as a new path that promoted connections with ourselves and with the wider society. 

## 5. Strengths and Limitations 

Dream sharing during stressful events in general and, specifically, during a pandemic is an understudied area of research; our study is, therefore, a significant contribution to the field. A qualitative exploration of the experiences of students offers a deeper understanding of the realities of both the COVID-19 pandemic and military conflict. Although COVID-19 is a global phenomenon, country-specific studies provide nuances of different experiences and add to the body of evidence of worldwide experiences [58]. More holistically, the current study adds to a growing body of science dealing with the dreamtelling approach and the impact of COVID-19.

Despite these contributions, the current study has several limitations. First, as a qualitative and exploratory one-session single case study, it was conducted in a specific cultural context and at a specific point in time. This limits the ability to generalize the results to wider populations. Second, the sample was unbalanced regarding the age, educational level, and academic affiliation of the participants. Our advertisement may, indeed, have attracted participants with a greater interest in dreams and psychology. 

These limitations notwithstanding, this case study encourages further research, both qualitative and quantitative, on group dreamtelling processes in other contexts, such as different settings, different conflicts, different cultures, and different populations. 

## 6. Conclusions

The results of this single case study that explored the experiences of the COVID-19 pandemic and local military conflict among a group of Israeli students using the dreamtelling approach enabled the exposure of profound depth structures and hidden themes as well as the digestion and relief of mental pain to be achieved through the creative social connections forged within the group. While no one is able to escape the impact of the ongoing pandemic, sharing it in a group can ease the burden and enhance coping possibilities. In line with this conclusion which supports the role of dreaming in the well-being, hope, and coping of individuals during periods of uncertainty in their waking life, we trust that our study can stimulate further psychological dream research, particularly concerning traumatic events, that focuses on the function of dreamtelling in facilitating affective integration and elaboration.

## Data Availability

The datasets used and analyzed in the current study are available upon request from the corresponding author.

## Appendix A. Codebook

**Table A1:** Category A: Interpretations.

No. of Code	Content	No. of Recurrences	No. of Speakers
1	Ability/lack of ability	7	5
2	Stuck/being blocked	5	3
3	Rocks and insecurity	4	2
4	Unsuccessful	5	3
5	Uncertainty	3	2
6	Leaving restricted area	3	2
7	Release/break out	3	2
8	Helplessness	3	2
9	Helplessness in the face of the known	2	2
10	No future	3	2
11	Longing for the known	2	2
12	Longing for certainty and security	2	2
12	Uniqueness/being separate	2	1
14	Growing up/not growing up	2	1
15	Leaving the comfort zone	2	2
16	Father	2	2
17	Steep path	2	1
18	Attempt to get to the beach	2	1
19	Presence of the other	2	1
20	Malice	1	1
21	Unknown and amorphous	1	1

**Table A2:** Category B: Emotional experiences.

No. of Code	Content	No. of Recurrences	No. of Speakers
1	Identification	3	3
2	Difficulty to break out	3	2
3	It felt like mine	2	2
4	It did not feel like mine	2	2
5	It took me to my childhood	2	2
6	Fear and worry	2	2
7	Positive warm feeling	1	1
8	Repetitious/ Sisyphean	2	2
9	Nausea	1	1
10	Someone does not protect me	1	1
11	The walls are closing in on me	1	1
12	Breaking in/Intrusion	1	1
13	No solution	2	1
14	Lost in an infinite equation	1	1
15	It feels like it’ll be OK	2	1

**Table A3:** Category C: Associations.

No. of Code	Content	No. of Recurrences	No. of Speakers
1	Difficult moments	2	2
2	Boulders	2	2
3	Childhood	2	2
4	Travels	2	2
5	Military conflict	2	2

**Table A4:** Category D: Associations with personal dreams.

No. of Code	Content	No. of Recurrences	No. of Speakers
1	Army/weapon	4	3
2	Helplessness	3	2
3	The walls are closing in on me	3	2
4	I didn’t manage to pull the trigger	2	2
5	Someone was breaking into the house	1	1
6	I couldn’t defend myself	1	1
7	Trying to shout and freeze	1	1

**Table A5:** Category E: Request for containment.

No. of Code	Content	No. of Recurrences	No. of Speakers
1	Containing the difficulty in the journey	5	4
2	Helping to complete the journey	3	2
3	Crossing together	2	2
4	Becoming subjects	2	2
5	Bypassing it together	2	1
6	Trolls	2	1
7	Transforming monsters to cute trolls	1	1
8	Containing helplessness	1	1
9	Opening up	1	1
10	Looking at the obstacle	1	1
11	Holding hands	1	1

**Table A6:** Category F: Being a member in the present group.

No. of Code	Content	No. of Recurrences	No. of Speakers
1	Pleasant	4	4
2	Partnership	4	3
3	Made me feel good/better	4	3
4	Comfortable	4	3
5	Enabling	2	2
6	Non-judgmental	2	2
7	Atmosphere of containment	2	2
8	Less comfortable	1	1
9	Belonging	1	1
10	A dance of the unconscious	1	1
